# Risk and protective factors associated with substance use among Puerto Rican youths after Hurricane María: a cross-sectional study

**DOI:** 10.1186/s12889-024-19720-2

**Published:** 2024-08-22

**Authors:** Juan Carlos Gonzalez, Daniel K. Feinberg, Regan W. Stewart, John Young, Rosaura Orengo-Aguayo

**Affiliations:** 1 Department of Psychiatry and Behavioral Sciences, University of California, San Francisco, San Francisco, CA USA; 2 Department of Counseling, Clinical, and School Psychology, University of California, Santa Barbara, CA USA; 3 Department of Psychiatry and Behavioral Sciences, Medical University of South Carolina, Charleston, SC USA; 4 Department of Psychology, University of Mississippi, MS, USA

**Keywords:** Substance use, Prevention, Natural disasters, Youth/adolescents

## Abstract

**Background:**

Identifying factors associated with post-disaster youth substance use is a crucial element of developing evidence-based prevention and intervention efforts. Hurricane María struck Puerto Rico in September of 2017 and the wide-spread impact from this disaster, including exposure to trauma, displacement, and disrupted social supports had the potential to negatively impact levels of substance use among youth across the archipelago. However, post-disaster substance use remains under-investigated in this context. The current study sought to identify risk and protective factors associated with substance use among Puerto Rican youth in the aftermath of Hurricane Maria.

**Methods:**

Cross-sectional, secondary data analyses were conducted using school-based survey data collected at all schools in Puerto Rico between February 1 and June 29, 2018 (5–9 months after Hurricane María). Social supports, substance use, and trauma symptoms were assessed. An ordinal regression analysis was conducted to identify student factors associated with greater likelihood of post-disaster substance use.

**Results:**

A total of 36,485 participants (50.7% female, grades 7–12), were included in an ordinal regression analysis that compared the likelihood of respondents endorsing high, low, or no substance use after Hurricane María based on reported adult social support, counselor/teacher social support, peer social support, ptsd symptomatology, and gender. Findings showed that, when compared to students that endorsed low or no substance use, those who reported having adult social support demonstrated a 58% reduction in odds (OR = 0.42, 95% CI: 0.34-0.53) of reporting high substance use after Hurricane María, while students who reported having teacher/counselor social support demonstrated a 21% reduction in odds (OR = 0.79, 95% CI: 0.69-0.89) of reporting high substance use. Additionally, those that reported having peer social support demonstrated a 31% increase in odds (OR = 1.31, 95% CI: 1.10 to 1.58) of reporting higher substance use, compared to those that reported low or no substance use.

**Conclusions:**

While social support was generally protective, prevention efforts to build positive family and community connections may be indicated. Evidence-based school screenings of substance use and trauma may help direct intervention to those most at risk for co-occurring issues.

**Supplementary Information:**

The online version contains supplementary material available at 10.1186/s12889-024-19720-2.

## Background

On September 20, 2017, Hurricane María struck the archipelago of Puerto Rico as a category four hurricane, wielding winds of over 150 mph and leaving upwards of 1.5 million people without electricity or potable water for three to twelve months in some regions [1]. Puerto Rico, a Caribbean nation typically characterized by its remarkable natural and human resources, witnessed unprecedented devastation to human life, public infrastructure, and land as a result of the hurricane. Natural disasters have been occurring at higher frequencies in recent years compared to previous decades due to climate change [2] and have subsequently put more youth at risk for developing mental health symptoms and disorders. Prior studies show that youth who have been exposed to disasters may develop new symptoms and those with pre-existing concerns may find that those concerns become exacerbated [3]. Previous research also suggests that post-traumatic stress symptoms in particular are common in disaster-exposed youth [4]. In fact, previous research found that 7.2% of youth met clinically-elevated levels of post-traumatic stress disorder (PTSD) symptoms following Hurricane María [5]. 

Despite being US citizens, Puerto Ricans living on the island prior to Hurricane María were at higher risk of experiencing substance use disorders (SUD), physical health problems (e.g., HIV, injection drug use), and poverty compared to mainland US residents [6–9]. Health and economic inequities, which have developed in part due to predatory legislative and financial policies, have perpetuated Puerto Rico’s *de facto* colonial status with the United States and limited access to crucial resources and services [1]. In addition, youth mental health needs are rising; 13-20% of youth meet criteria for a mental health disorder in the U.S. in any given year [10] with alcohol and substance use disorders among the most prevalent [11] and the most costly [12]. In studies of Puerto Rican youth specifically, higher rates of substance use have been associated with other risky behaviors and involvement with the juvenile justice system [13]. Thus, Hurricane María added additional stressors for Puerto Rican youth who were already at elevated risk for developing alcohol and substance use behaviors when compared with youth in the U.S. mainland.

Extensiveness of disaster exposure is a primary risk factor for mental health symptomatology in youth populations [14]. Previous research examining post-disaster substance use among adolescents has identified greater loss of services/resources and PTSD symptomatology as primary risk factors [15]. While research has demonstrated a strong link between traumatic natural disasters and increased rates of substance abuse by affected youth, it has also been shown that specific protective factors can make a difference in encouraging healthy development in spite of such challenges [16]. Commonly cited protective factors associated with less substance use, in general, include positive parental influence [17, 18], prosocial social supports [19], and positive bonds to community and school [20]. However, alcohol and substance use in response to natural disasters remain understudied in youth. The limited extant data available suggest that disaster exposure is associated with increased substance use for youth in the months immediately following disasters [15] and may have longitudinal implications for elevated use later in life when disaster exposure occurred at a young age [16, 21]. Given so few studies in this domain, comparisons across various ethnocultural variables have not been possible and minimal research to date has documented the prevalence of post-disaster substance use in a primarily Hispanic and Caribbean sample. This is a significant gap given the inequities that exist within this population in comparison to the rest of the United States and the strong likelihood that disasters similar to Hurricane María will occur again in the Caribbean region and others. In fact, during the preparation of this manuscript yet another hurricane, Hurricane Fiona, made landfall on the Puerto Rican archipelago, leading to damaged crops, mudslides, and over 30 inches of rainfall in some regions [22]. Thus, the current study has two aims: (1) to identify of the risk and protective factors associated with differing levels of substance use among youth who were exposed to Hurricane María in Puerto Rico and (2) to propose evidence-based recommendations for how to best mobilize resources and services when a similar disaster occurs in the future. In line with previous literature, our study team hypothesized that post-disaster substance use would be negatively associated with social support and positively associated with trauma symptomatology.

## Methods

### Participants

The current study presents a secondary analysis of data collected by Orengo-Aguayo and colleagues [5] in partnership with the Puerto Rico Department of Education. Given that the typical age of onset for substance use occurs after age 12 [23], a subset of the original dataset (*n* = 96,108) including only 7th-12th grade students that were missing no data needed for the current analyses was examined for the present study (*n* = 36,485). Participants that were excluded from current analyses did not differ significantly from those included except with regard to age and completeness of survey data. Please see Table 1 for a breakdown of all observed characteristics of participants included in the present study.

**Table 1:** Participant characteristics by substance use level

Substance Use Level				
Characteristic	N	None, *N* = 34,075^1^	Low, *N* = 1,741^1^	High, *N* = 669^1^
**Region**	36,485			
Arecibo		1,906 (5.59%)	71 (4.08%)	21 (3.14%)
Bayamon		4,598 (13.49%)	214 (12.29%)	60 (8.97%)
Caguas		4,455 (13.07%)	274 (15.74%)	95 (14.20%)
Humacao		5,285 (15.51%)	282 (16.20%)	133 (19.88%)
Mayaguez		6,421 (18.84%)	303 (17.40%)	104 (15.55%)
Ponce		6,811 (19.99%)	348 (19.99%)	131 (19.58%)
San Juan		4,599 (13.51%)	249 (14.30%)	125 (18.68%)
Missing values		0	0	0
**Grade**	36,485			
Grade 7		6,609 (19.40%)	104 (5.97%)	41 (6.13%)
Grade 8		6,422 (18.85%)	146 (8.39%)	52 (7.77%)
Grade 9		5,500 (16.14%)	222 (12.75%)	85 (12.71%)
Grade 10		5,476 (16.07%)	304 (17.46%)	106 (15.84%)
Grade 11		5,468 (16.05%)	415 (23.84%)	160 (23.92%)
Grade 12		4,600 (13.49%)	550 (31.59%)	225 (33.63%)
Missing values		0	0	0
**Gender**	36,485			
Male		16,694 (48.99%)	860 (49.40%)	415 (62.03%)
Female		17,381 (51.01%)	881 (50.60%)	254 (37.97%)
Missing values		0	0	0
**PTSD Total**	36,485	7.272 (6.48)	11.573 (7.61)	12.833 (9.53)
Missing values		0	0	0
**Social Support Total**	36,485			
0 of 3 Endorsed		668 (1.96%)	54 (3.10%)	45 (6.73%)
1 of 3 Endorsed		2,930 (8.60%)	189 (10.86%)	82 (12.26%)
2 of 3 Endorsed		12,667 (37.17%)	742 (42.62%)	281 (42.00%)
3 of 3 Endorsed		17,810 (52.27%)	756 (43.42%)	261 (39.01%)
Missing values		0	0	0
**Adult SS**	36,485			
No		1,570 (4.61%)	188 (10.80%)	101 (15.10%)
Yes		32,505 (95.39%)	1,553 (89.20%)	568 (84.90%)
Missing values		0	0	0
**Peer SS**	36,485			
No		4,252 (12.48%)	205 (11.77%)	113 (16.89%)
Yes		29,823 (87.52%)	1,536 (88.23%)	556 (83.11%)
Missing values		0	0	0
**Teacher SS**	36,485			
No		14,709 (43.17%)	889 (51.06%)	366 (54.71%)
Yes		19,366 (56.83%)	852 (48.94%)	303 (45.29%)
Missing values		0	0	0

^1^n (%); Mean (SD)

### Measures

#### Hurricane Maria-related trauma exposure and PTSD symptoms

The present study utilized a modified version of the National Center for Child Traumatic Stress Network and Hurricane Assessment and Referral Tool (NCTSN-HART) [24]. The modified version consisted of items from the Hurricane-Related Traumatic Experiences-Revised Scale [25] and the University of California Los Angeles Posttraumatic Stress Disorder Reaction Index-Brief (UCLA-RI Brief; [26]), as well as supplementary items that assessed social support and drug or alcohol use that were added in consultation with the Puerto Rico Department of Education. Item language was adapted to match the verbiage used in Puerto Rico. The final form of the instrument was decided upon after close communication between one of the authors of the current paper, a bilingual Puerto Rican native to the island, and the Puerto Rico Department of Education. Participants were given the option to take the survey in Spanish or English. Constructs measured included exposure to hurricane-related trauma, post-traumatic stress symptoms, social supports, and substance use.

##### Hurricane-related trauma

Participants were administered 19 “yes” or “no” items from the NCTSN-HART that measured perceived life threat, actual life threat, past loss and disruption, and ongoing loss and disruption that may have resulted from the effects of Hurricane María’s landfall in Puerto Rico.

##### PTSD symptomatology

Participants were administered 12 items from the UCLA-RI Brief that measured symptoms included in DSM-5 PTSD Criteria B (intrusions), C (avoidance), D (negative alterations in cognition/mood), and E (alterations in arousal/reactivity). Responses could be scored via the following 5-point Likert-type scale: 0: “Not at all”, 1: “A little”, 2: “Somewhat”, 3: “Quite a bit”, 4: “Very much”, NA: “Don’t know.” Scores across the 12 items were summed for each participant and each participant was given a total PTSD symptom score. The present sample demonstrated an internal consistency (Cronbach’s 𝛼 = 0.79) for the UCLA-RI Brief that was acceptable.

##### Substance use

One survey item was presented to all participants inquiring about substance use (i.e., “Have you used drugs or alcohol since Hurricane Maria?”). This item served as the dependent variable of the ordinal logistic regression used in the present study. Substance use was reported via the following 5-point Likert-type scale: 0: “Not at all”, 1: “A little”, 2: “Somewhat”, 3: “Quite a bit”, 4: “Very much”, NA: “Don’t know.” Due to the low percentage of participants endorsing any substance use (see Table 1), ratings of substance use were collapsed into three categories: no substance use (i.e., responses of 0), low substance use (i.e., responses of 1’s and 2’s), or high substance use (i.e., responses of 3’s and 4’s). Responses of “Don’t know” were not included in analyses.

##### Social supports

Surveys included three items to rate social supports. Participants were asked, “Do you have any adult in your life (like your parents, family member) in whom you can trust and know they will be there for you?”, “Do you have any friend in your life in whom you can trust and know they will be there for you?”, “Do you have any teacher or counselor in your life in whom you can trust and know they will be there for you?” Participants could indicate “yes,” or “no,” for each of the three social support items.

### Procedures

Ethics approval was received collaboratively by the Medical University of South Carolina and the Puerto Rico Department of Education. The original data collection occurred through a needs assessment facilitated by the Puerto Rico Department of Education, who waived the requirement for parental written consent given the anonymous nature of the data collection. All study procedures were deemed exempt from the need for full IRB approval by the Medical University of South Carolina’s IRB due to the minimal risk involved for participants and the anonymous nature of data collection; therefore, the IRB at the Medical University of South Carolina waived the requirement to obtain consent from individual participants in accordance with federal regulations. The Puerto Rico Department of Education sent survey instruments to every public school in Puerto Rico that was open at the time of survey implementation. Several schools were still closed at that time due to the effects of Hurricane Maria, and their students were assigned to other area schools. However, not all schools chose to participate in the survey. Although not all schools responded to the survey, schools who participated represented all 5 educational regions across the archipelago. Representatives from participating public schools (*N* = 1,086) were supplied with packets that included paper surveys to be distributed to students, as well as instructions for their administration and collection. School representatives distributed paper surveys to teachers who then administered the surveys to their students between five and nine months after Hurricane María made landfall. Teachers administered the paper and pencil-based survey to students in classrooms, soliciting verbal assent, indicating that children could opt-out at any time without penalty, and ensuring that no identifying information appeared on response sheets. After surveys were completed, school representatives delivered them to a central location where processing was conducted by the Puerto Rico Department of Education. Please see Orengo-Aguayo and colleagues [5]for further detail regarding data collection procedures.

### Data analytic plan

#### Software

All analyses were conducted using the R Statistical language (version 4.2.2) via Rstudio (version 2022.12.0) on Windows 10 × 64 (build 22621), using the following packages: ordinal (version 2022.11.16), AER (version 1.2.10), gtsummary (version 1.7.0), MASS (version 7.3.58.1), tidyverse (version 1.3.2), dplyr (version 1.0.10), and rcompanion (version 2.4.18). Code for the existing project can be found below in the Declarations.

#### Model, criteria, and goodness of fit

A multivariable general ordinal logistic regression model was conducted to identify which student factors were associated with greater likelihood of post-disaster substance use. Specifically, the model examined the likelihood of a given student to self-report no substance use, a low level of substance use, or a high level of substance use based on factors such as adult social support (yes/no), peer social support (yes/no), teacher/counselor social support (yes/no), total PTSD symptomatology (range: 0–48), grade level (range: 7–12), and gender (binary). Interaction terms for gender and PTSD symptomatology, gender and adult social support, gender and peer social support, and gender and teacher/counselor social support were also included in the model. Results of the analysis were examined in terms of statistical significance, as well as effect size. All tests were conducted using an alpha level of *p* < 0.05 as the criterion for significance. The variable coefficients produced by the ordinal logistic regression apply equally to the discrimination between the likelihood of participants’ endorsement of the category of *no use* versus *low use* and the likelihood of participants endorsement of *low use* versus *high use.*

Goodness-of-fit for the model was determined by comparing it to a null model using the Nagelkerke pseudo R-squared and likelihood ratio tests. The log likelihood ratio Chi-Square test yielded a significant result (LR χ² [14] = 2033.7, *p* < 0.001), suggesting that the model with the predictor variables provided a substantially better fit than a null model. The Nagelkerke pseudo R² value for the model was 0.125 and McFadden’s pseudo-R² value was computed to be 0.099. These results indicated that the full model explained significantly more of the variance in the dependent variable when compared to the null model. In particular, McFadden’s R-squared in particular was indicative of the robustness of the model given the complexity and number of predictors involved. Based on these results, it appears that the model’s overall effect size is substantial and its results are worth consideration. Please see Table 2 for a variable-by-variable breakdown of the model’s results.

**Table 2:** Ordinal logistic regression table

Variables	Log Odds	OR^1^	OR 95% CI^2^	SE^2^
**Adult Social Support**	-0.865***	0.42***	**(0.34 to 0.53)**	0.108
**Peer Social Support**	0.273**	1.31**	**(1.10 to 1.58)**	0.092
**Teacher Social Support**	-0.242***	0.79***	**(0.69 to 0.89)**	0.063
**PTSD Symptom Total**	0.086***	1.09***	**(1.08 to 1.10)**	0.004
**Grade Level**	0.388***	1.47***	**(1.43 to 1.52)**	0.014
**Gender**				
Male	—	—		—
Female	-0.195	0.82	(0.58 to 1.17)	0.179
PTSD Total * Gender				
PTSD Total * Female	-0.015**	0.99**	**(0.98 to 1.00)**	0.005
Adult SS * Gender				
Adult SS * Female	-0.023	0.98	(0.73 to 1.32)	0.152
Peer SS * Gender				
Peer SS * Female	-0.034	0.97	(0.73 to 1.28)	0.141
Teacher SS * Gender				
Teacher SS * Female	-0.049	0.95	(0.80 to 1.14)	0.091
**Threshold Coefficients**				
No Use | Low Use	6.386	593.27	(414.96 to 848.20)	0.182
Low Use | High Use	7.785	2404.17	(1669.20 to 3462.76)	0.186
Number of Obs.	36,485			
McFadden Pseudo-R²	0.099			
Cox and Snell (ML)	0.054			
Nagelkerke	0.125			

^1^ *p < 0.05; **p < 0.01; ***p < 0.001

^2^ OR = Odds Ratio, CI = Confidence Interval, SE = Standard Error

Results in bold indicate statistically significant findings

## Results

### Ordinal logistic regression

#### Main effects

With all other variables held constant, the model’s results predicted that students who endorsed having adult social support, compared to those who did not, demonstrated a 58% reduction in odds of endorsing a higher category of substance use after Hurricane María (OR = 0.42, 95% CI: 0.34-0.53). Students that reported having peer social support, compared to those who did not, demonstrated a 33% increase in odds of endorsing a higher category of substance use (OR = 1.33), while students who indicated that they had teacher/ counselor social support demonstrated a 21% reduction in odds (OR = 0.79, 95% CI: 0.69-0.89), compared to students who did not have teacher/counselor support. Additionally, for each one-point increase in PTSD symptomatology reported, students’ odds of endorsing a higher category of substance use increased by approximately 9% (OR = 1.09, 95% CI: 1.10 to 1.58). Lastly, students in higher grades demonstrated an increase in odds of endorsing a higher category of substance use such that, per each grade level increase, the odds of having endorsing a higher substance use category increased 47% (OR = 1.47, 95% CI: 1.43 to 1.52).

#### Interactions

 A significant interaction was evidenced between PTSD symptomatology and gender, suggesting that females with higher levels of PTSD exhibited a 1% lower likelihood of reporting a higher category of substance use compared to males with similar levels of PTSD (OR = 0.99, 95% CI: 0.98 to 1.00). While statistically significant, this 1% difference in likelihood has limited practical application, as it is likely a result of the large sample size included in the model. Other interactions involving adult, peer, and teacher social support by gender were not significant, indicating no differential impact of these forms of social support on substance use by gender.

A likelihood ratio test was conducted to compare the fit of the full model against a model without the significant interaction between PTSD symptomatology and gender. The results of the likelihood ratio test indicated a statistically significant improvement in model fit (LR χ² [1] = 7.72, *p* < 0.01) when the interaction term was included. Thus, the model suggests that the effect of PTSD symptoms on substance use is affected by gender and the inclusion of the interaction effect in the model provided a significantly better fit to the data than the model that did not include the interaction.

#### Thresholds

The thresholds for transitioning from None to Low Substance Use (Estimate = 6.39, SE = 0.18, z = 35.01) and from Low to High substance use (Estimate = 7.79, SE = 0.19, z = 41.82) were significant, supporting the ordinal nature of the substance use variable in this sample.

## Discussion

### Summary of findings

The current study utilized a large sample of Puerto Rican youths from grades seven through twelve to identify predictors of substance use in the months following Hurricane María. This study adds to burgeoning literature examining post-disaster substance use specifically in a primarily Hispanic and Caribbean sample of school-aged youth. Our main findings indicate that while more social support is generally protective against youth substance use following natural disasters, not all social support has the same positive impact on youth. Specifically, trusting relationships with an adult, teacher, or counselor were positively indicated as protective factors against substance use in the wake of Hurricane María. In line with previous research that suggests trusted adult influence can be protective against youth substance use in general [19], our findings show that both adults at home and teachers within the school can serve this positive role. Recent studies show that family cohesion [27] and school connectedness [28] can both have a positive influence on youth developmental and social outcomes, including substance use. Our study suggests that adults, both at home and/or at school, can be key sources of support during the months immediately following natural disasters.

On the contrary, respondents’ endorsement of having a trusted peer was associated with greater likelihood for substance use following Hurricane María. Potentially contradicting research that suggests prosocial peer contact can serve as a protective factor against youth substance use [19], our study indicated that having a trusted peer could also be predictive of negative outcomes. This is potentially consistent with previous findings that youth are more likely to use substances with peers than alone, particularly when those peers engage in unhealthy behaviors [29, 30]. The current study did not obtain more detailed information concerning the nature of social interactions or the propensity to facilitate greater exposure to substance use. This could be a valuable area for future research, particularly given some research has found that greater peer approval of substance use positively influences the likelihood of self-reported substance use among Puerto Rican youth [31]. The quality of peer relationships and the ways in which substances are used socially are both likely to have an influence on the likelihood that any given youth would turn to substance use following a disaster.

In addition to social support, higher grade and PTSD symptomatology were also significantly associated with differences in substance use endorsement. Grade findings were expected given well-established trends of substance use trajectories that indicate a general increase from early to late adolescence [23]. Although cross-sectional and subject to potential cohort effects, the patterns of escalating use noted in the current data were consistent with these previous findings and reflective of the importance of targeted prevention at earlier stages of development in order to curtail later use. This may be particularly important in under-resourced, disenfranchised, minoritized populations, particularly those affected by natural disasters. However, more research will be necessary to understand differential risk across cultural variables. What can be more clearly elucidated in connection to existing studies is that PTSD symptoms commonly co-occurs with problematic substance use in both adults and youth [32, 33], which was supported by the current findings. However, the detailed nature of the relationship between these variables remains to be investigated; further research should seek to understand the contexts in which substances are being used and their potential relationship to coping with trauma-related symptoms [34]. Advances in understanding potential etiological risk for post-disaster substance use, particularly in terms of traumatic stress incurred as a result of the disaster, could help direct future prevention and interventions efforts in Puerto Rico and elsewhere. This is especially salient for the archipelago of Puerto Rico, given the very strong likelihood of future occurrences of similar disasters and the historically limited, untimely response of the US government in providing aid, re-establishing basic utilities, and/or repairing basic infrastructure.

### Limitations and suggestions for future research

While estimates of island-wide youth and adolescent substance use are somewhat outdated, existing reports suggest that 12-month prevalence rates of alcohol and drug use among adolescents in Puerto Rico (without disorder symptoms) previously stood at 29.6% and 19.0% respectively [35]. This highlights that our sample could generally be under-reporting substance use in the months following Hurricane María, perhaps due to social desirability [36], a form of self-report bias, generally exhibited when data is collected in less private formats (e.g., classrooms). It is also possible that limitations or strain on resources immediately following the hurricane (including school closures), may have influenced reporting behaviors. A second notable limitation of the current dataset is our inability to distinguish between various types of peer social supports. While our interpretation of the data suggests that peer support was associated with higher likelihood for substance use in the wake of natural disasters, the variable used to understand peer influence was not sensitive or varied enough to measure different kinds of peer influence (i.e., prosocial vs. negative impact). Future studies would benefit from utilizing student reports to differentiate prosocial and negative peer influences on substance use following natural disasters [19]. In addition, substance use cut-offs were based on reported frequency of use but did not capture intensity. This measurement does not allow for as sensitive a look at potential impact or taxonomic implication (i.e., the degree to which an individual met criteria for a substance use disorder following the hurricane). Similarly, without differentiation of specific substances used in the wake of natural disasters, we were unable to determine who might have been at highest risk for worst-case outcomes such as overdose or death. It is important to note, however, that the priority of our team was to meet the needs of our partner, the Puerto Rico Department of Education, during a highly stressful and trying time period (5 or more months post-Hurricane María), and to ensure this needs assessment was short, easy to administer in self-report format, and did not overburden the already taxed school systems in this post-disaster context. Finally, future research should include longitudinal design and study of similar outcomes, given that the cross-sectional design does not allow for causal inference between variables measured in the current study.

## Conclusions

Given the wide-ranging negative impacts that youth substance use and disaster exposure separately can have on youth well-being, understanding their intersection is critical to promoting positive outcomes for youth that experience either or both. As climate change leads to greater frequency and intensity of natural disasters [2], the current study’s findings can be mobilized to inform prevention and intervention efforts in the inevitability of another hurricane in the Caribbean. Based on the present study’s findings, the relationship between PTSD symptoms and higher likelihood of high substance use following disaster is in need of further investigation. Utilizing longitudinal methods, future studies should and aim to identify the ways in which youth are using substances following disaster in order to direct treatment efforts. Meanwhile, trauma and substance use screeners, particularly when collected in partnership with local schools or community-based organizations, may be helpful tools in surveying those at risk prior to a natural disaster occurring. Screening would be particularly helpful in identifying those at risk for negative outcomes of compound symptomatology (e.g., PTSD and substance use; 34). Given the identified link between positive adult influence and lower risk of high substance use in the current sample, it is possible that intervention efforts focused on developing positive family and community connections (e.g., school and community connectedness) could promote prosocial coping before and after natural disasters. Findings suggesting the potentially conflicting impact of peer relationships may suggest that interventions to enhance peer relationships could be secondary to those outlined above, at least without more research and attention to ensuring the prosocial nature of peer interactions. Overall, the current study highlights important areas of overlap with existing research. The findings add to a growing body of literature that informs evidence-based decision making to promote resilience against trauma and healthy development in youth.

### Electronic supplementary material

Below is the link to the electronic supplementary material.

Supplementary Material 1

## Data Availability

The data that support the findings of this study were received from the Puerto Rico Department of Education, but restrictions apply to the availability of these data. The data were used under agreement and provided for the use of the current study, and so are not publicly available. However, code for the existing project is publicly available at the following hyperlink: https://github.com/daniel-k-feinberg/gonzalez-et-al-2024.
