# Supplementary material for: Risk and protective factors associated with substance use among Puerto Rican youths after Hurricane María: a cross-sectional study
Source: BMC Public Health. 2024 Aug 22;24:2291. doi: 10.1186/s12889-024-19720-2 (PMC11342666; doi:10.1186/s12889-024-19720-2)
Supplement: Supplementary file 1 — Supplementary Material 1 [file 12889_2024_19720_MOESM1_ESM.pdf]

STUDENTS
(Adapted from the NCTSN Hurricane Assessment Tool by MUSC team)

School Code : ☐☐☐☐☐

Grade: 12 ○ 11 ○ 10 ○ 9 ○ 8 ○ 7 ○ 6 ○ 5 ○ 4 ○ 3 ○ 2 ○ 1 ○ K ○

Gender: M ○ | F ○

ParteA. The following questions ask about your experience during and after **Hurricane María**. Circle in the corresponding column.

	Question	Yes	No
1.	Were you injured during Hurricane María?	○	○
2.	Was any family member, neighbor, or friend injured during the hurricane?	○	○
3.	Did any family member, neighbor, or friend die as a result of the hurricane?	○	○
4.	Did you think you could die during Hurricane María?	○	○
5.	Was your house destroyed or greatly damaged (for example: roof top flew away, walls crumbled, windows break, water came inside) as a result of the hurricane?	○	○
6.	Were your clothes, toys, or any other belongings damaged as a result of the hurricane?	○	○
7.	Did you have to evacuate your home and stay at a safer place (for example: a shelter, a family member or neighbor's home) as a result of the hurricane?	○	○
8.	Are you still living in another place that is not your home (for example: a shelter, family member or neighbor's house) as a result of the hurricane?	○	○
9.	Did you see houses or other places around you destroyed or greatly damaged (for example: flooding, destroyed homes or bridges destroyed bridges, fallen light posts) as a result of the hurricane?	○	○
10.	Did you loose a pet (for example: your dog or cat) because it got stranded, passed away, or had to give it to someone else as a result of the hurricane?	○	○
11.	Has your family struggled to find food or water after the hurricane?	○	○
12.	Have things been stolen from your home or in your neighborhood since the hurricane?	○	○
13.	Have you seen fights or violence in your home or neighborhood since the hurricane?	○	○
14.	Did you move to a different school (different to the one you use to attend before the hurricane) as a result of the hurricane?	○	○
15.	Did you help to save or rescue people during the hurricane?	○	○
16.	Did any of your parents lose their job as a result of the hurricane?	○	○
17.	Has electricity been restored (did the lights come back) where you live?	○	○
18.	Has water service been restored where you live?	○	○
19.	Do you have any friends or family members that have moved to the mainland US after the hurricane?	○	○
20.	Do you have any **adult** in your life (like your parents, family member) in whom you can trust and know they will be there for you?	○	○
21.	Do you have any **friend** in your life in whom you can trust and know they will be there for you?	○	○
22.	Do you have any **teacher or counselor in your life** in whom you can trust and know they will be there for you?	○	○

STUDENTS
(Adapted from the *NCTSN Hurricane Assessment Tool* by MUSC team)

Part B: We want to know how you have been feeling since **Hurricane María**. Think about your thoughts, feelings, and behavior DURING THE LAST MONTH. Use the guide below to show how often you have had this problem during the last month.

		Not at all (0)	A Little (1)	Somewhat (2)	Quite a Bit (3)	Very Much (4)	Don't Know
1	Do you try to stay away from people, places, or things that remind you of hurricane Maria?	○	○	○	○	○	○
2	Do you get upset easily or get into arguments or physical fights?	○	○	○	○	○	○
3	Do you have trouble concentrating or paying attention?	○	○	○	○	○	○
4	When something reminds you of the hurricane, do you get very upset, afraid, or sad?	○	○	○	○	○	○
5	Do you have trouble feeling happiness or love?	○	○	○	○	○	○
6	Do you try not to think about or have feelings about the hurricane?	○	○	○	○	○	○
7	When something reminds you of the hurricane, do you have strong feelings in your body, like your heart beats fast, your head aches, or stomach aches?	○	○	○	○	○	○
8	Do you have thoughts like, "I will never be able to trust other people"?	○	○	○	○	○	○
9	Do you feel alone even when you are around other people?	○	○	○	○	○	○
10	Do you have upsetting thoughts, pictures, or sounds of the hurricane come into your mind when you don't want them to?	○	○	○	○	○	○
11	Do you have trouble going to sleep, wake up often, or have trouble getting back to sleep?	○	○	○	○	○	○
12	Do you worry about something else bad happening to you/your family/your friends?	○	○	○	○	○	○
13	Do you feel very sad, down, or depressed?	○	○	○	○	○	○
14	Do you have less energy than usual?	○	○	○	○	○	○
15	If in school: Do you find it harder to get your schoolwork done? after the hurricane?	○	○	○	○	○	○
16	Are you having a harder time getting along with your family or your friends after the hurricane?	○	○	○	○	○	○
17	If in a new school: Are you having a hard time making new friends?	○	○	○	○	○	○
18	Are you finding it harder to do or enjoy activities that you used to enjoy?	○	○	○	○	○	○
19	Have you used drugs or alcohol since the hurricane?	○	○	○	○	○	○
